# 2-Bromo-*N*′-[(*Z*)-2-bromo­benzyl­idene]-5-methoxy­benzohydrazide

**DOI:** 10.1107/S1600536809024921

**Published:** 2009-07-04

**Authors:** Hongqi Li, B. K. Sarojini, B. Narayana, H. S. Yathirajan, William T. A. Harrison

**Affiliations:** a Key Laboratory of Science & Technology of Eco-Textiles, Ministry of Education, College of Chemistry, Chemical Engineering & Biotechnology, Donghua University, Shanghai 201620, People’s Republic of China; b Department of Chemistry, P. A. College of Engineering, Mangalore 574 153, India; c Department of Studies in Chemistry, Mangalore University, Mangalagangotri 574 199, India; d Department of Studies in Chemistry, University of Mysore, Manasagangotri, Mysore 570 006, India; e Department of Chemistry, University of Aberdeen, Aberdeen AB24 3UE, Scotland

## Abstract

In the title compound, C_15_H_12_Br_2_N_2_O_2_, the mol­ecule adopts an *E* conformation about the C=N double bond and a *transoid* conformation about the central N—N bond, with a C(=O)—N—N—C(H) dihedral angle of 169.4 (4)°. In the crystal, mol­ecules are linked by N—H⋯O hydrogen bonds, leading to *C*(4) chains. The packing also features slipped π–π stacking inter­actions, with a centroid–centroid separation of 3.838 (3) Å and a slippage of 1.19 Å.

## Related literature

For related structures and background, see: Narayana *et al.* (2007 ▶); Butcher *et al.* (2007 ▶).
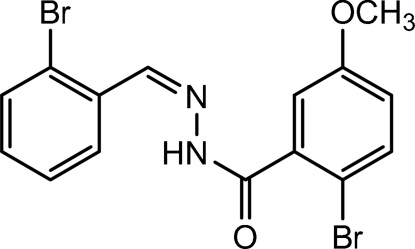


## Experimental

### 

#### Crystal data

C_15_H_12_Br_2_N_2_O_2_
*M* *_r_* = 412.09
Monoclinic, 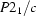

*a* = 14.768 (5) Å
*b* = 12.753 (4) Å
*c* = 8.227 (3) Å
β = 96.114 (4)°
*V* = 1540.6 (9) Å^3^
*Z* = 4
Mo *K*α radiation
μ = 5.27 mm^−1^
*T* = 296 K
0.30 × 0.20 × 0.20 mm

#### Data collection

Bruker SMART CCD area-detector diffractometer
Absorption correction: multi-scan (*SADABS*; Bruker, 2006 ▶) *T*_min_ = 0.301, *T*_max_ = 0.419 (expected range = 0.251–0.349)
9369 measured reflections
3515 independent reflections
1902 reflections with *I* > 2σ(*I*)
*R*_int_ = 0.063

#### Refinement

*R*[*F* ^2^ > 2σ(*F* ^2^)] = 0.047
*wR*(*F* ^2^) = 0.122
*S* = 0.96
3515 reflections
195 parameters
H atoms treated by a mixture of independent and constrained refinement
Δρ_max_ = 0.57 e Å^−3^
Δρ_min_ = −0.81 e Å^−3^

### 

Data collection: *SMART* (Bruker, 2006 ▶); cell refinement: *SAINT* (Bruker, 2006 ▶); data reduction: *SAINT*; program(s) used to solve structure: *SHELXS97* (Sheldrick, 2008 ▶); program(s) used to refine structure: *SHELXL97* (Sheldrick, 2008 ▶); molecular graphics: *SHELXTL* (Sheldrick, 2008 ▶); software used to prepare material for publication: *SHELXTL*.

## Supplementary Material

Crystal structure: contains datablocks global, I. DOI: 10.1107/S1600536809024921/fj2231sup1.cif

Structure factors: contains datablocks I. DOI: 10.1107/S1600536809024921/fj2231Isup2.hkl

Additional supplementary materials:  crystallographic information; 3D view; checkCIF report

## Figures and Tables

**Table 1:** Hydrogen-bond geometry (Å, °)

*D*—H⋯*A*	*D*—H	H⋯*A*	*D*⋯*A*	*D*—H⋯*A*
N1—H1⋯O1^i^	0.87 (4)	2.07 (4)	2.906 (4)	160 (4)

Symmetry code: (i) 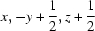.

**Fig. 1.**
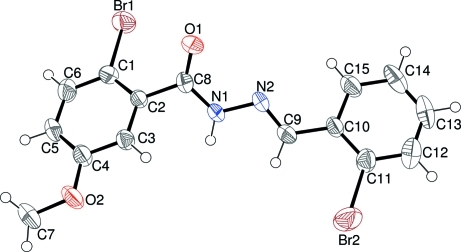
A view of (I) with displacement ellipsoids for the non-hydrogen atoms drawn at the 50% probability level.

**Fig. 2.**
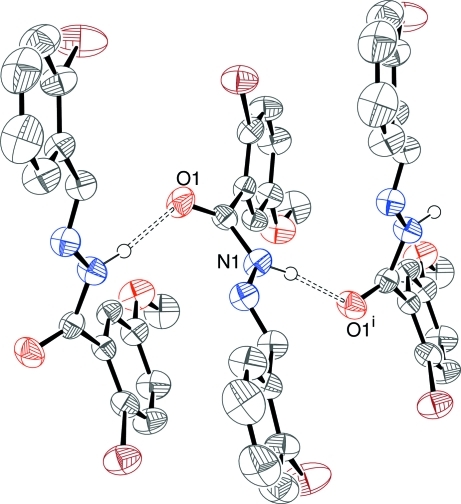
A fragment of an [001] C(4) chain of molcules in the crystal of (I). Symmetry code as in Table 2.

## supplementary crystallographic information

### Comment

As part of our ongoing studies of substituted benzohydrazides (Narayana *et al.*,  2007; Butcher *et al.*, 2007) we now describe the synthesis and crystal structure of the title compound, (I) (Fig. 1).

The dihedral angle between the mean planes of the A (C1–C6) and B (C10–C15) rings is 18.6 (3)°. Atom C7 is dispalaced from the A plane by 0.064 (9)Å.  The molecule is significantly twisted about the N1—N2 bond.

In the crystal, an intermolecular N—H···O interaction occurs (Table 2), leading to C(4) chains (Fig. 2) of molecules propagating in [001]. The shortest intermolecular aromatic ring centroid–centroid separation is 3.638 (3)Å, indicative of weak π-π stacking.

### Experimental

A mixture of 2-bromobenzaldehyde (1.85 g, 0.01 mol) and 2-bromo-5-methoxybenzo-hydrazide (2.45 g, 0.01mol) in 15 ml of ethanol containing 2 drops of 4 M hydrochloric acid was refluxed for 3 hours. On cooling, the solid separated was filtered and recrystallized from ethyl alcohol to yield colourless blocks of (I) (m.p: 440-442 K).  Analysis (%) for C_15_H_12_Br_2_N_2_O_2_: calculated (found): C 43.73 (43.66), H 2.94 (2.91), N 6.80 (6.76).

### Refinement

The N-bound H atom was located in a difference map and its position was freely refined.   All the other H atoms were placed in idealized locations (C—H = 0.93–0.98 Å) and refined as riding with *U*_iso_(H) = 1.2*U*_eq_(C,N) or 1.5*U*_eq_(methyl C). The methyl group was allowed to rotate, but not to tip, to best fit the electron density.

### Crystal data

**Table:**

C_15_H_12_Br_2_N_2_O_2_	*F*(000) = 808
*M**_r_* = 412.09	*D*_x_ = 1.777 Mg m^−^^3^
Monoclinic, *P*2_1_/*c*	Melting point = 440–442 K
Hall symbol: -P 2ybc	Mo *K*α radiation, λ = 0.71073 Å
*a* = 14.768 (5) Å	Cell parameters from 1776 reflections
*b* = 12.753 (4) Å	θ = 2.8–22.8°
*c* = 8.227 (3) Å	µ = 5.27 mm^−^^1^
β = 96.114 (4)°	*T* = 296 K
*V* = 1540.6 (9) Å^3^	Block, colourless
*Z* = 4	0.30 × 0.20 × 0.20 mm

### Data collection

**Table:**

Bruker SMART CCD area-detector diffractometer	3515 independent reflections
Radiation source: fine-focus sealed tube	1902 reflections with *I* > 2σ(*I*)
graphite	*R*_int_ = 0.063
φ and ω scans	θ_max_ = 27.5°, θ_min_ = 2.1°
Absorption correction: multi-scan (*SADABS*; Bruker, 2006)	*h* = −19→14
*T*_min_ = 0.301, *T*_max_ = 0.419	*k* = −12→16
9369 measured reflections	*l* = −10→10

### Refinement

**Table:**

Refinement on *F*^2^	Secondary atom site location: difference Fourier map
Least-squares matrix: full	Hydrogen site location: inferred from neighbouring sites
*R*[*F*^2^ > 2σ(*F*^2^)] = 0.047	H atoms treated by a mixture of independent and constrained refinement
*wR*(*F*^2^) = 0.122	*w* = 1/[σ^2^(*F*_o_^2^) + (0.0504*P*)^2^] where *P* = (*F*_o_^2^ + 2*F*_c_^2^)/3
*S* = 0.96	(Δ/σ)_max_ = 0.001
3515 reflections	Δρ_max_ = 0.57 e Å^−^^3^
195 parameters	Δρ_min_ = −0.81 e Å^−^^3^
0 restraints	Extinction correction: *SHELXL97* (Sheldrick, 2008), Fc^*^=kFc[1+0.001xFc^2^λ^3^/sin(2θ)]^-1/4^
Primary atom site location: structure-invariant direct methods	Extinction coefficient: 0.0145 (10)

### Special details

**Table:**

Geometry. All esds (except the esd in the dihedral angle between two l.s. planes) are estimated using the full covariance matrix. The cell esds are taken into account individually in the estimation of esds in distances, angles and torsion angles; correlations between esds in cell parameters are only used when they are defined by crystal symmetry. An approximate (isotropic) treatment of cell esds is used for estimating esds involving l.s. planes.
Refinement. Refinement of F^2^ against ALL reflections. The weighted R-factor wR and goodness of fit S are based on F^2^, conventional R-factors R are based on F, with F set to zero for negative F^2^. The threshold expression of F^2^ > 2sigma(F^2^) is used only for calculating R-factors(gt) etc. and is not relevant to the choice of reflections for refinement. R-factors based on F^2^ are statistically about twice as large as those based on F, and R- factors based on ALL data will be even larger.

### Fractional atomic coordinates and isotropic or equivalent isotropic displacement parameters (Å^2^)

**Table:**

	*x*	*y*	*z*	*U*_iso_*/*U*_eq_	
C1	0.1558 (3)	0.0150 (3)	0.5766 (5)	0.0405 (11)	
C2	0.1498 (3)	0.1213 (3)	0.5376 (5)	0.0322 (9)	
C3	0.0842 (3)	0.1808 (3)	0.6039 (5)	0.0367 (10)	
H3	0.0794	0.2520	0.5796	0.044*	
C4	0.0256 (3)	0.1365 (3)	0.7055 (5)	0.0415 (11)	
C5	0.0317 (3)	0.0314 (4)	0.7407 (6)	0.0536 (13)	
H5	−0.0084	0.0006	0.8065	0.064*	
C6	0.0973 (3)	−0.0282 (4)	0.6780 (6)	0.0522 (12)	
H6	0.1024	−0.0990	0.7045	0.063*	
C7	−0.0958 (3)	0.1633 (4)	0.8725 (6)	0.0655 (15)	
H7A	−0.1332	0.1093	0.8189	0.098*	
H7B	−0.1339	0.2188	0.9054	0.098*	
H7C	−0.0608	0.1347	0.9672	0.098*	
C8	0.2084 (3)	0.1719 (3)	0.4227 (5)	0.0356 (10)	
C9	0.3247 (3)	0.4087 (4)	0.4401 (5)	0.0406 (10)	
H9	0.2985	0.4355	0.5293	0.049*	
C10	0.3904 (3)	0.4723 (3)	0.3608 (5)	0.0374 (10)	
C11	0.3967 (3)	0.5806 (4)	0.3780 (6)	0.0477 (11)	
C12	0.4617 (4)	0.6382 (4)	0.3079 (7)	0.0646 (15)	
H12	0.4641	0.7107	0.3198	0.077*	
C13	0.5223 (4)	0.5874 (5)	0.2207 (7)	0.0712 (17)	
H13	0.5668	0.6254	0.1742	0.085*	
C14	0.5179 (4)	0.4814 (5)	0.2017 (7)	0.0708 (16)	
H14	0.5590	0.4475	0.1415	0.085*	
C15	0.4530 (3)	0.4241 (4)	0.2707 (6)	0.0527 (13)	
H15	0.4510	0.3517	0.2568	0.063*	
Br1	0.24530 (3)	−0.07259 (4)	0.49919 (6)	0.0578 (2)	
Br2	0.31365 (4)	0.65524 (4)	0.49486 (9)	0.0861 (3)	
O1	0.2174 (2)	0.1358 (2)	0.2869 (3)	0.0489 (8)	
O2	−0.0358 (2)	0.2037 (3)	0.7627 (4)	0.0579 (9)	
N1	0.2468 (2)	0.2623 (3)	0.4809 (4)	0.0400 (9)	
H1	0.239 (3)	0.277 (3)	0.582 (5)	0.048*	
N2	0.3036 (2)	0.3173 (3)	0.3872 (4)	0.0391 (9)	

### Atomic displacement parameters (Å^2^)

**Table:**

	*U*^11^	*U*^22^	*U*^33^	*U*^12^	*U*^13^	*U*^23^
C1	0.038 (3)	0.042 (3)	0.042 (3)	−0.002 (2)	0.011 (2)	−0.007 (2)
C2	0.030 (2)	0.037 (2)	0.030 (2)	−0.0044 (18)	0.0070 (18)	−0.0026 (18)
C3	0.038 (2)	0.036 (2)	0.038 (2)	0.0012 (19)	0.0104 (19)	0.0061 (19)
C4	0.036 (2)	0.046 (3)	0.044 (3)	0.000 (2)	0.012 (2)	0.003 (2)
C5	0.056 (3)	0.051 (3)	0.060 (3)	−0.010 (2)	0.034 (3)	0.004 (3)
C6	0.056 (3)	0.038 (3)	0.066 (3)	−0.005 (2)	0.020 (3)	0.008 (2)
C7	0.051 (3)	0.080 (4)	0.071 (4)	−0.002 (3)	0.035 (3)	0.000 (3)
C8	0.035 (2)	0.037 (3)	0.035 (2)	−0.0032 (19)	0.0082 (19)	0.007 (2)
C9	0.037 (2)	0.044 (3)	0.043 (3)	0.003 (2)	0.017 (2)	−0.001 (2)
C10	0.033 (2)	0.039 (3)	0.042 (2)	0.0010 (19)	0.0143 (19)	0.006 (2)
C11	0.037 (2)	0.043 (3)	0.064 (3)	−0.002 (2)	0.011 (2)	0.006 (2)
C12	0.057 (3)	0.056 (3)	0.080 (4)	−0.014 (3)	0.006 (3)	0.019 (3)
C13	0.055 (3)	0.091 (5)	0.070 (4)	−0.022 (3)	0.020 (3)	0.026 (3)
C14	0.051 (3)	0.100 (5)	0.068 (4)	0.001 (3)	0.035 (3)	0.012 (3)
C15	0.048 (3)	0.056 (3)	0.058 (3)	0.005 (2)	0.027 (2)	0.004 (2)
Br1	0.0569 (4)	0.0486 (3)	0.0713 (4)	0.0116 (2)	0.0227 (3)	−0.0006 (2)
Br2	0.0675 (4)	0.0502 (4)	0.1467 (7)	0.0080 (3)	0.0393 (4)	−0.0194 (4)
O1	0.066 (2)	0.050 (2)	0.0337 (17)	−0.0067 (16)	0.0203 (15)	−0.0084 (15)
O2	0.0502 (19)	0.059 (2)	0.071 (2)	0.0080 (17)	0.0370 (17)	0.0058 (18)
N1	0.044 (2)	0.045 (2)	0.035 (2)	−0.0083 (17)	0.0204 (18)	−0.0031 (18)
N2	0.039 (2)	0.044 (2)	0.038 (2)	−0.0050 (17)	0.0188 (16)	0.0016 (17)

### Geometric parameters (Å, °)

**Table:**

C1—C6	1.378 (6)	C8—N1	1.350 (5)
C1—C2	1.394 (6)	C9—N2	1.272 (5)
C1—Br1	1.892 (4)	C9—C10	1.469 (6)
C2—C3	1.387 (5)	C9—H9	0.9300
C2—C8	1.495 (5)	C10—C15	1.388 (6)
C3—C4	1.386 (5)	C10—C11	1.390 (6)
C3—H3	0.9300	C11—C12	1.382 (6)
C4—O2	1.367 (5)	C11—Br2	1.894 (5)
C4—C5	1.373 (6)	C12—C13	1.369 (8)
C5—C6	1.375 (6)	C12—H12	0.9300
C5—H5	0.9300	C13—C14	1.361 (8)
C6—H6	0.9300	C13—H13	0.9300
C7—O2	1.427 (5)	C14—C15	1.375 (7)
C7—H7A	0.9600	C14—H14	0.9300
C7—H7B	0.9600	C15—H15	0.9300
C7—H7C	0.9600	N1—N2	1.388 (4)
C8—O1	1.229 (5)	N1—H1	0.87 (4)
			
C6—C1—C2	120.0 (4)	N2—C9—C10	120.3 (4)
C6—C1—Br1	118.2 (3)	N2—C9—H9	119.9
C2—C1—Br1	121.8 (3)	C10—C9—H9	119.9
C3—C2—C1	118.2 (3)	C15—C10—C11	116.9 (4)
C3—C2—C8	119.2 (4)	C15—C10—C9	120.1 (4)
C1—C2—C8	122.6 (3)	C11—C10—C9	122.9 (4)
C4—C3—C2	121.4 (4)	C12—C11—C10	121.9 (4)
C4—C3—H3	119.3	C12—C11—Br2	117.4 (4)
C2—C3—H3	119.3	C10—C11—Br2	120.7 (3)
O2—C4—C5	124.8 (4)	C13—C12—C11	119.2 (5)
O2—C4—C3	115.6 (4)	C13—C12—H12	120.4
C5—C4—C3	119.6 (4)	C11—C12—H12	120.4
C4—C5—C6	119.6 (4)	C14—C13—C12	120.3 (5)
C4—C5—H5	120.2	C14—C13—H13	119.9
C6—C5—H5	120.2	C12—C13—H13	119.9
C5—C6—C1	121.2 (4)	C13—C14—C15	120.5 (5)
C5—C6—H6	119.4	C13—C14—H14	119.7
C1—C6—H6	119.4	C15—C14—H14	119.7
O2—C7—H7A	109.5	C14—C15—C10	121.2 (5)
O2—C7—H7B	109.5	C14—C15—H15	119.4
H7A—C7—H7B	109.5	C10—C15—H15	119.4
O2—C7—H7C	109.5	C4—O2—C7	118.1 (4)
H7A—C7—H7C	109.5	C8—N1—N2	119.4 (3)
H7B—C7—H7C	109.5	C8—N1—H1	115 (3)
O1—C8—N1	124.1 (4)	N2—N1—H1	125 (3)
O1—C8—C2	122.7 (4)	C9—N2—N1	114.5 (3)
N1—C8—C2	113.2 (4)		
			
C6—C1—C2—C3	0.2 (6)	N2—C9—C10—C11	160.4 (4)
Br1—C1—C2—C3	−177.8 (3)	C15—C10—C11—C12	0.5 (7)
C6—C1—C2—C8	−177.3 (4)	C9—C10—C11—C12	177.2 (4)
Br1—C1—C2—C8	4.7 (6)	C15—C10—C11—Br2	178.8 (3)
C1—C2—C3—C4	−0.3 (6)	C9—C10—C11—Br2	−4.4 (6)
C8—C2—C3—C4	177.2 (4)	C10—C11—C12—C13	−0.8 (8)
C2—C3—C4—O2	180.0 (4)	Br2—C11—C12—C13	−179.2 (4)
C2—C3—C4—C5	−0.6 (7)	C11—C12—C13—C14	0.8 (8)
O2—C4—C5—C6	−179.0 (4)	C12—C13—C14—C15	−0.5 (9)
C3—C4—C5—C6	1.7 (7)	C13—C14—C15—C10	0.2 (8)
C4—C5—C6—C1	−1.8 (8)	C11—C10—C15—C14	−0.2 (7)
C2—C1—C6—C5	0.9 (7)	C9—C10—C15—C14	−177.0 (5)
Br1—C1—C6—C5	179.0 (4)	C5—C4—O2—C7	2.8 (7)
C3—C2—C8—O1	−127.5 (4)	C3—C4—O2—C7	−177.8 (4)
C1—C2—C8—O1	50.0 (6)	O1—C8—N1—N2	−2.9 (6)
C3—C2—C8—N1	50.2 (5)	C2—C8—N1—N2	179.5 (3)
C1—C2—C8—N1	−132.4 (4)	C10—C9—N2—N1	174.9 (4)
N2—C9—C10—C15	−23.0 (7)	C8—N1—N2—C9	169.4 (4)

### Hydrogen-bond geometry (Å, °)

**Table:**

*D*—H···*A*	*D*—H	H···*A*	*D*···*A*	*D*—H···*A*
N1—H1···O1^i^	0.87 (4)	2.07 (4)	2.906 (4)	160 (4)

Symmetry codes: (i) *x*, −*y*+1/2, *z*+1/2.
